# Distribution of lipid parameters according to different socio-economic indicators- the EPIC-Norfolk prospective population study

**DOI:** 10.1186/1471-2458-14-782

**Published:** 2014-08-28

**Authors:** Shamarina Shohaimi, Matthijs S Boekholdt, Robert Luben, Nick J Wareham, Kay-Tee Khaw

**Affiliations:** Department of Biology, Faculty of Science, Universiti Putra Malaysia, Serdang, Selangor 43400 Malaysia; Department of Cardiology, Academic Medical Center, Amsterdam, The Netherlands; Department of Public Health and Primary Care, University of Cambridge, Cambridge, UK; MRC Epidemiology Unit, University of Cambridge School of Clinical Medicine, Cambridge, UK

## Abstract

**Background:**

Data on the relationship between plasma levels of cholesterol and triglycerides and social class have been inconsistent. Most previous studies have used one classification of social class.

**Methods:**

This was a cross-sectional population based study with data on occupational social class, educational level obtained using a detailed health and lifestyle questionnaire. A total of 10,147 men and 12,304 women aged 45–80 years living in Norfolk, United Kingdom, were recruited using general practice age-sex registers as part of the European Prospective Investigation into Cancer (EPIC-Norfolk). Plasma levels of cholesterol and triglycerides were measured in baseline samples. Social class was classified according to three classifications: occupation, educational level, and area deprivation score according to Townsend deprivation index. Differences in lipid levels by socio-economic status indices were quantified by analysis of variance (ANOVA) and multiple linear regression after adjusting for body mass index and alcohol consumption.

**Results:**

Total cholesterol levels were associated with occupational level among men, and with educational level among women. Triglyceride levels were associated with educational level and occupational level among women, but the latter association was lost after adjustment for age and body mass index. HDL-cholesterol levels were associated with both educational level and educational level among men and women. The relationships with educational level were substantially attenuated by adjustment for age, body mass index and alcohol use, whereas the association with educational class was retained upon adjustment. LDL-cholesterol levels were not associated with social class indices among men, but a positive association was observed with educational class among women. This association was not affected by adjustment for age, body mass index and alcohol use.

**Conclusions:**

The findings of this study suggest that there are sex differences in the association between socio-economic status and serum lipid levels. The variations in lipid profile with socio-economic status may be largely attributed to potentially modifiable factors such as obesity, physical activity and dietary intake.

## Background

Coronary heart disease (CHD) is the leading cause of death in the world. Elevated cholesterol levels are a major risk factor for CHD. In developed countries, socio-economic differences in CHD mortality have been reported consistently [1, 2]. However, the association between socio-economic status and cholesterol level has been less consistent. Several studies have observed inverse relationships between socio-economic status and cholesterol levels [3–5], others have reported higher cholesterol levels among those with higher education or higher-grade of employment [6–8] while some found no association at all [9]. In developing countries the association is often positive with those in higher socio-economic status having higher levels of serum lipids [10–12]. In most of these studies, socio-economic status was usually measured using only one indicator of socio-economic status, mainly educational level. However, in order to capture the full extent of the influence of socio-economic status, the use of several measures of socio-economic status may be more appropriate. This is because different indicators of socio-economic status measure different dimensions, which may influence health status differently [6–13]. In the present study we investigated the cross-sectional association between three different measures of socio-economic status at both individual and area-level and the four lipid parameters commonly used in clinical practice: total cholesterol, high-density lipoprotein cholesterol (HDL-C), low-density lipoprotein cholesterol (LDL-C) and triglycerides in a population-based study of men and women living in Norfolk, United Kingdom.

## Methods

The study population was based in Norfolk, United Kingdom. A total of 77,630 men and women aged between 39–79 were identified from age-sex registers from general practices and were invited by mail to participate in the baseline survey [14]. The cohort was part of the European Prospective Investigation into Cancer (EPIC-Norfolk), which was designed to investigate the aetiology of major chronic diseases. A total of 30,445 agreed to participate (45% response rate) and gave informed consent and completed a detailed health and lifestyle questionnaire. Of these, 25,639 attended a health check. The response rate of 45% is not uncommon in a large population-based studies [15, 16] and studies have shown that underlying response rates do not bias exposure-outcome relationships [17, 18]. Detailed descriptions of the recruitment and study methodology have been reported previously [14]. The study was approved by the Norfolk District Ethics Committee and all participants gave signed informed consent.

### Anthropometic, lifestyle and socio-economic measurements

Body mass index (BMI) was estimated as weight (kg)/ height (m)^2^. Participants’ height and weight were measured with participants in light clothing and with their shoes removed. Height was measured to the nearest 0.1 cm using a stadiometer while weight was measured to the nearest 100 g using Salter scales. All measurements were made by trained nurses using standardised methods. Alcohol consumption (in grams per day) was derived from the food frequency questionnaire [19]. Participants were asked to indicate how often on average they drank beer, liquors and spirits during the past year prior to the survey. If a person drank 2 glasses of beer per day and 2 glasses of whisky per week on average, the food frequency box 2–3 per day for beer and 2–4 per week for spirits would be marked. Alcohol consumption was calculated by multiplying the frequency of alcohol consumed by standard portion weights to obtain grams of alcohol consumed per day. Socio-economic status was measured using three different indices: social class by occupation, educational level and area deprivation score. Details of each measure are described elsewhere [20]. Briefly, information on occupation and educational level were obtained from the health and lifestyle questionnaire. Social class was classified according to the registrar general’s occupation-based classification scheme into five main categories, with social class I representing professional classes and social class V representing groups such as manual labourers [21]. For men, social class was coded using their current occupation at the time of survey except when they were unemployed or retired in which case their partner’s social class was used. Unemployed men without partners were unclassified. Social class in women was based on their partner’s except when the partner’s social class was unclassified, missing or if they had no partner in which case social class was based on their own occupation. An unemployed woman without a partner was coded as unclassified. Educational status was based on the highest qualification attained and was categorised into four groups: degree or equivalent, A-level or equivalent, O-level or equivalent and less than O-level or no qualifications. O-level indicates educational attainment to the equivalent of completion of schooling to the age of 15 years and A-level indicates educational attainment to the equivalent of completion of schooling to the age of 17 years. Residential area-deprivation was measured using the Townsend Deprivation Index [22]. Participants were attributed to a 1991 census enumeration district based on their postcodes at time of survey. Using variables derived from the census, the Townsend score was generated for each enumeration district as a measure of material deprivation. The participants were then grouped into quintiles of Townsend deprivation index by their score.

### Biochemical analyses

Non-fasting blood samples were obtained by venepuncture as previously described [14]. Serum concentrations of total cholesterol, high-density lipoprotein cholesterol (HDL) and triglycerides were measured with the RA 1000 Technicon analyser (Bayer Diagnostics, Basingstoke) while low-density lipoprotein cholesterol (LDL) concentration was calculated using the Friedewald formula except when triglyceride concentration was more than 4 mmol/L.

### Statistical analyses

Analyses were undertaken for men and women separately. Concentrations of total cholesterol, HDL-C, LDL-C and triglycerides were tabulated according to the three measures of socio-economic status: occupational social class, level of education and deprivation level. They were then adjusted for possible confounders body mass index and alcohol consumption. Differences in lipid levels by socio-economic status indices were quantified by analysis of variance (ANOVA) and linear regression. We categorised social class, educational level and area-based deprivation as dichotomous variables. Social classes I, II and III non-manual were classified as “non-manual”, while social classes III manual, IV and V were classified as “manual”. Educational level was categorised into “at least O-level” (which includes O-level, A-level and degree) and “no qualifications”. For residential deprivation, subjects with Townsend scores of less than 0 were classified as “less deprived”, while those with Townsend scores of more than 0 were categorised as “most deprived”. P-values < 0.05 were considered statistically significant. Statistical analyses were performed using SPSS Version 19.0 (SPSS Inc., Chicago, Illinois).

#### Ethical approval

The study was approved by the Norfolk District Ethics Committee and all participants gave signed informed consent.

## Results

### Total cholesterol

Women had slightly higher mean total cholesterol levels than men (Table 1). Among men, total cholesterol levels were not associated with socio-economic status using any of the three classification methods. When the three indicators of socio-economic status were dichotomized, total cholesterol level was significantly lower in men with non-manual profession compared to men with a manual profession (Table 2), even after adjustment for age and BMI. Total cholesterol was not associated with education or deprivation level. Among women, total cholesterol levels were not related with occupation-based class or deprivation category. There was, however, a significant trend towards higher total cholesterol level among women with lower educational level (p < 0.001). This relationship persisted after adjustment for age and BMI (p = 0.004). This relationship was explained entirely by a higher total cholesterol level among people without qualification compared to those with any qualification, whereas there was no trend observed across different levels of qualification. Total cholesterol was higher among those with at least O-level compared to those without qualifications (Table 2). Among women, total cholesterol level was not associated with profession or deprivation level.

**Table 1:** **Mean total cholesterol and triglyceride levels at baseline survey by social class, educational level and deprivation category**

	Men					Women				
	N = 10,147					N = 12,304				
	*Total cholesterol*			*Triglycerides*		*Total cholesterol*			*Triglycerides*	
Social class	*n*	*	†	*	†	n	*	†	*	†
I	782	6.03	6.05	1.89	1.92	802	6.24	6.26	1.48	1.51
II	3888	6.03	6.03	1.88	1.88	4352	6.27	6.27	1.51	1.53
III Non-manual	1259	6.00	6.00	1.87	1.87	2464	6.24	6.24	1.53	1.54
III Manual	2570	5.95	5.95	1.86	1.85	2574	6.27	6.26	1.59	1.57
IV	1354	5.98	5.98	1.89	1.88	1640	6.27	6.25	1.59	1.56
V	294	5.99	5.98	1.93	1.93	472	6.37	6.34	1.65	1.59
*p value for trend*		*0.1*	*0.06*	*0.8*	*0.3*		*0.3*	*0.6*	*<0.001*	*0.05*
**Educational level**										
Degree or equivalent	1571	6.01	6.02	1.84	1.88	1357	6.22	6.24	1.46	1.49
A-level or equivalent	4636	6.01	6.01	1.86	1.86	3234	6.22	6.22	1.49	1.49
O-level or equivalent	882	6.03	6.03	1.92	1.92	2011	6.24	6.24	1.50	1.52
No qualifications	3059	5.97	5.96	1.90	1.88	5701	6.31	6.30	1.61	1.60
*p value for trend*		*0.2*	*0.1*	*0.05*	*0.2*		*<0.001*	*0.004*	*<0.001*	*<0.001*
**Deprivation category** ^‡^										
1 (<−3.80)	2068	6.00	6.00	1.86	1.88	2466	6.28	6.28	1.52	1.53
2 (−3.79 to −2.92)	2139	5.99	5.99	1.87	1.86	2542	6.26	6.26	1.53	1.53
3 (−2.91 to −2.09)	1971	6.02	6.02	1.86	1.86	2368	6.25	6.25	1.53	1.54
4 (−2.08 to −0.55)	2042	5.98	5.97	1.89	1.88	2517	6.25	6.24	1.57	1.55
5 (> − 0.54)	1927	6.00	6.00	1.90	1.90	2411	6.28	6.27	1.58	1.57
*p value for trend*		*0.8*	*0.7*	*0.4*	*0.5*		*0.9*	*0.8*	*0.01*	*0.2*

^‡^Based on Townsend deprivation scores.

*Adjusted for age.

^**†**^Adjusted for age and body mass index.

**Table 2:** **Regression coefficients for mean total cholesterol level for models based on social class, level of education and deprivation level**

Men	Age-adjusted		Age- and BMI-adjusted	
	Regression coefficient (95% CI)	*p value*	Regression coefficient (95% CI)	*p value*
Social class (Manual vs non-manual^‡^)	−0.055 (−0.10 to −0.01)	*0.01*	−0.058 (−0.10 to 0.01)	*0.01*
Education (No qualifications vs at least O-level^‡^)	−0.031 (−0.079 to 0.18)	*0.2*	−0.038 (−0.086 to 0.01)	*0.1*
Deprivation level (Highly deprived vs less deprived^‡^)	0.054 (−0.004 to 0.11)	*0.07*	0.054 (−0.003 to 0.11)	*0.07*
**Women**	**Age-adjusted**		**Age- and BMI-adjusted**	
	**Regression coefficient (95% CI)**	***p value***	**Regression coefficient (95% CI)**	***p value***
Social class (Manual vs non-manual^‡^)	−0.002 (−0.043 to −0.04)	*0.9*	−0.022 (−0.064 to 0.02)	*0.3*
Education (No qualifications vs at least O-level^‡^)	0.085 (0.044 to 0.13)	*< 0.001*	0.077 (0.035 to 0.12)	*< 0.001*
Deprivation level (Highly deprived vs less deprived^‡^)	0.023 (−0.03 to 0.08)	*0.4*	0.021 (−0.032 to 0.07)	*0.4*

Regression coefficients are shown as the difference in mmol/L from the reference category, adjusted for the other factors in the model.

^‡^Reference category.

Predictor variables: Social class- non-manual = social classes I, II and III non-manual, manual = social classes III manual, IV and V.

Education- at least O level, no qualifications.

Deprivation level- based on Townsend deprivation scores: < 0 = less deprived, > 0 = highly deprived.

### Triglycerides

Triglyceride levels were higher among men than women (Table 1). Among men, triglyceride levels were not associated with occupational class or deprivation category. There was evidence for an inverse relationship between triglyceride levels and education level, but this relationship was entirely explained by age and BMI differences. Triglyceride levels were also not associated with socio-economic status after dichotomization (Table 3). Among women, triglyceride levels were significantly associated with lower socio-economic status according to all three classifications. These relationships were attenuated by adjustment for age and BMI, but remained statistically significant for occupational class and educational level. After adjustment for age and BMI, the relationship between triglycerides and deprivation category lost statistical significance. When parameters of socio-economic status were dichotomized, triglycerides were significantly associated with education level, even after adjustment for age and BMI (Table 3). The relationship between professional status and triglycerides was entirely explained by the difference in BMI.

**Table 3:** **Regression coefficients for mean triglyceride levels for models based on social class, level of education and deprivation level**

MEN	Age-adjusted		Age- and BMI-adjusted	
	Regression coefficient (95% CI)	*p value*	Regression coefficient (95% CI)	*p value*
Social class (Manual vs non-manual^‡^)	−0.020 (−0.57- 0.18)	*0.30*	−0.027 (−0.063- 0.009)	*0.14*
Education (No qualifications vs at least O-level^‡^)	0.039 (−0.002- 0.08)	*0.06*	0.017 (−0.02- 0.06)	*0.39*
Deprivation level (Highly deprived vs less deprived^‡^)	0.043 (−0.006- 0.092)	*0.09*	0.043 (−0.004- 0.09)	*0.07*
**Women**	**Age-adjusted**		**Age- and BMI-adjusted**	
	**Regression coefficient (95% CI)**	***p value***	**Regression coefficient (95% CI)**	***p value***
Social class (Manual vs non-manual^‡^)	0.055 (0.027- 0.083)	*< 0.001*	0.014 (−0.014- 0.041)	*0.33*
Education (No qualifications vs at least O-level^‡^)	0.11 (0.079- 0.14)	*< 0.001*	0.09 (0.063- 0.12)	*< 0.001*
Deprivation level (Highly deprived vs less deprived^‡^)	0.034 (−0.002- 0.07)	*0.06*	0.031 (−0.004- 0.07)	*0.08*

Regression coefficients are shown as the difference in mmol/L from the reference category, adjusted for the other factors in the model.

^‡^Reference category.

Predictor variables: Social class- non-manual = social classes I, II and III non-manual, manual = social classes III manual, IV and V.

Education- at least O level, no qualifications.

Deprivation level- based on Townsend deprivation scores: < 0 = less deprived, > 0 = highly deprived.

### HDL-cholesterol

Women had higher HDL-cholesterol levels than men (Tables 4 and 5). Among men, HDL-cholesterol levels were significantly positively associated with occupational class and educational level. These associations persisted upon adjustment for age and BMI, but lost statistical significance upon additional adjustment for alcohol use. Among men, HDL-cholesterol levels were not associated with deprivation category. When parameters of socio-economic status were dichotomized, HDL-cholesterol levels were not associated with occupational class or deprivation score (Table 6). HDL-cholesterol levels were significantly lower among men with at least O-level education compared to men without qualifications. Part of this relationship was explained by differences in BMI levels and alcohol use. Among women, HDL-cholesterol levels were also significantly positively associated with occupational class and educational level. These relationships remained significant after adjustment for age and BMI, and also upon additional adjustment for alcohol use. Among women, HDL-cholesterol levels were also not associated with deprivation category. HDL-cholesterol levels were significantly lower among women with at least O-level education compared to women without qualifications, even after adjustment for age, BMI and alcohol use. The relationship between HDL-cholesterol and occupational class and deprivation level were not statistically significant.

**Table 4:** **Mean HDL-cholesterol and LDL-cholesterol levels at baseline survey by social class, educational level and deprivation category**

	Men						
	N = 10,147						
	HDL-cholesterol				LDL-cholesterol		
Social class	*n*	*	†	‡	*	†	‡
I	782	1.26	1.25	1.24	3.93	3.94	3.93
II	3888	1.25	1.25	1.23	3.94	3.94	3.93
III Non-manual	1259	1.23	1.23	1.23	3.92	3.92	3.92
III Manual	2570	1.21	1.22	1.23	3.90	3.90	3.90
IV	1354	1.22	1.23	1.24	3.91	3.90	3.91
V	294	1.23	1.23	1.25	3.88	3.88	3.88
*p value for trend*		*0.001*	*0.01*	*0.8*	*0.7*	*0.6*	*0.8*
**Educational level**							
Degree or equivalent	1571	1.26	1.25	1.24	3.92	3.92	3.92
A-level or equivalent	4636	1.24	1.24	1.24	3.93	3.93	3.93
O-level or equivalent	882	1.23	1.23	1.23	3.94	3.94	3.93
No qualifications	3059	1.20	1.21	1.22	3.90	3.90	3.90
*p value for trend*		*<0.001*	*<0.001*	*0.09*	*0.7*	*0.6*	*0.7*
**Deprivation category** ^**‡**^							
1 (<−3.80)	2068	1.24	1.23	1.23	3.92	3.93	3.93
2 (−3.79 to −2.92)	2139	1.23	1.23	1.24	3.92	3.92	3.92
3 (−2.91 to −2.09)	1971	1.23	1.23	1.23	3.95	3.95	3.95
4 (−2.08 to −0.55)	2042	1.23	1.23	1.24	3.90	3.89	3.89
5 (> − 0.54)	1927	1.24	1.24	1.23	3.91	3.91	3.92
*p value for trend*		*0.8*	*1.0*	*1.0*	*0.6*	*0.5*	*0.5*

^‡^Based on Townsend deprivation scores.

*Adjusted for age.

**†**Adjusted for age and body mass index.

**‡**Adjusted for age, body mass index and alcohol intake.

**Table 5:** **Mean HDL-cholesterol and LDL-cholesterol levels at baseline survey by social class, educational level and deprivation category**

	Women						
	N = 12,304						
	HDL-cholesterol				LDL-cholesterol		
Social class	*n*	*	†	‡	*	†	‡
I	802	1.65	1.63	1.61	3.94	3.96	3.98
II	4352	1.60	1.60	1.58	3.99	3.99	4.01
III Non-manual	2464	1.56	1.56	1.56	3.99	4.00	3.99
III Manual	2574	1.52	1.53	1.55	4.03	4.02	4.01
IV	1640	1.53	1.55	1.56	4.02	4.00	3.99
V	472	1.51	1.54	1.57	4.11	4.08	4.06
*p value for trend*		*<0.001*	*<0.001*	*0.002*	*0.04*	*0.4*	*0.8*
**Educational level**							
Degree or equivalent	1357	1.65	1.64	1.61	3.92	3.93	3.95
A-level or equivalent	3234	1.60	1.60	1.59	3.95	3.95	3.96
O-level or equivalent	2011	1.58	1.57	1.57	3.98	3.99	3.99
No qualifications	5701	1.52	1.53	1.54	4.06	4.05	4.04
*p value for trend*		*<0.001*	*<0.001*	*<0.001*	*<0.001*	*<0.001*	*0.001*
**Deprivation category** ^**‡**^							
1 (<−3.80)	2466	1.57	1.56	1.56	4.03	4.03	4.04
2 (−3.79 to −2.92)	2542	1.58	1.58	1.58	4.00	4.00	4.00
3 (−2.91 to −2.09)	2368	1.56	1.56	1.56	4.00	4.01	4.01
4 (−2.08 to −0.55)	2517	1.56	1.57	1.57	3.98	3.98	3.98
5 (> − 0.54)	2411	1.57	1.58	1.58	4.00	3.99	3.99
*p value for trend*		*0.4*	*0.3*	*0.3*	*0.7*	*0.4*	*0.4*

^‡^Based on Townsend deprivation scores.

*Adjusted for age.

**†**Adjusted for age and body mass index.

**‡**Adjusted for age, body mass index and alcohol intake.

**Table 6:** **Regression coefficients for mean HDL-cholesterol for models based on social class, level of education and deprivation level**

Men	Age-adjusted		Age- and BMI-adjusted		Age-, BMI-, and alcohol-adjusted	
	Regression coefficient (95% CI)	*p value*	Regression coefficient (95% CI)	*p value*	Regression coefficient (95% CI)	*p value*
Social class (Manual vs non-manual^‡^)	−0.017 (−0.03- -0.003)	*0.02*	−0.015 (−0.028- 0.001)	*0.03*	0.002 (−0.01- 0.02)	*0.8*
Education (No qualifications vs at least O-level^‡^)	−0.036 (−0.05- -0.02)	*<0.001*	−0.029 (−0.04- -0.01)	*<0.001*	−0.016 (−0.03- -0.002)	*0.03*
Deprivation level (Highly deprived vs less deprived^‡^)	−0.008 (−0.01- 0.03)	*0.4*	−0.008(−0.009- 0.03)	*0.4*	−0.001 (−0.02- 0.02)	*0.9*
**Women**	**Age-adjusted**		**Age- and BMI-adjusted**		**Age-, BMI- and alcohol-adjusted**	
	**Regression coefficient (95% CI)**	***p value***	**Regression coefficient (95% CI)**	***p value***	**Regression coefficient (95% CI)**	***p value***
Social class (Manual vs non-manual^‡^)	−0.053 (−0.07- -0.04)	*<0.001*	−0.033 (−0.049- -0.02)	*<0.001*	−0.012 (−0.028- 0.004)	*0.1*
Education (No qualifications vs at least O-level^‡^)	−0.068 (−0.08- -0.05)	*<0.001*	−0.059 (−0.08- -0.04)	*<0.001*	−0.042 (−0.058-0.03)	*<0.001*
Deprivation level (Highly deprived vs less deprived^‡^)	0.017 (−0.004- 0.04)	*0.1*	0.018 (−0.002-0.04)	*0.08*	−0.017 (−0.003- 0.04)	*0.09*

Regression coefficients are shown as the difference in mmol/L from the reference category, adjusted for the other factors in the model. ^‡^Reference category. Predictor variables: Social class- non-manual = social classes I, II and III non-manual, manual = social classes III manual, IV and V. Education- at least O level, no qualifications. Deprivation level- based on Townsend deprivation scores: < 0 = less deprived, > 0 = highly deprived.

### LDL-cholesterol

LDL-cholesterol levels were higher among women than men (Tables 4 and 5). Among men, LDL-cholesterol levels were associated with neither occupational class, nor with educational level nor with deprivation category. These relationships were also absent when socio-economic status was dichotomized (Table 7). Among women, LDL-cholesterol levels were significantly inversely associated with educational level, but not with occupational level or with deprivation category. The relationship between LDL-cholesterol level and educational level retained statistical significance even upon adjustment for age, BMI and alcohol use. LDL-cholesterol levels were significantly lower among women with at least O-level education compared to women without qualifications.

**Table 7:** **Regression coefficients for mean LDL-cholesterol for models based on social class, level of education and deprivation level**

Men	Age-adjusted		Age- and BMI-adjusted		Age-, BMI- and alcohol-adjusted	
	Regression coefficient (95% CI)	*p value*	Regression coefficient (95% CI)	*p value*	Regression coefficient (95% CI)	*p value*
Social class (Manual vs non-manual^‡^)	−0.03 (−0.07-0.01)	*0.1*	−0.031 (−0.071- 0.008)	*0.1*	−0.027 (−0.067- 0.01)	*0.2*
Education (No qualifications vs at least O-level^‡^)	−0.017 (−0.06- 0.03)	*0.5*	−0.02 (−0.063- 0.02)	*0.4*	−0.016 (−0.06- 0.03)	*0.5*
Deprivation level (Highly deprived vs less deprived^‡^)	0.025 (−0.027- 0.08)	*0.4*	0.026 (−0.026- 0.08)	*0.3*	0.024 (−0.029- 0.08)	*0.4*
**Women**	**Age-adjusted**		**Age- and BMI-adjusted**		**Age-, BMI- and alcohol-adjusted**	
	**Regression coefficient (95% CI)**	***p value***	**Regression coefficient (95% CI)**	***p value***	**Regression coefficient (95% CI)**	***p value***
Social class (Manual vs non-manual^‡^)	0.025 (−0.014- 0.06)	*0.2*	0.002 (−0.037- 0.04)	*0.9*	−0.014 (−0.053- 0.03)	*0.5*
Education (No qualifications vs at least O-level^‡^)	0.099 (0.06- 0.14)	*<0.001*	0.09 (0.051- 0.13)	*<0.001*	0.077 (0.038- 0.12)	*<0.001*
Deprivation level (Highly deprived vs less deprived^‡^)	−0.005 (−0.055- 0.04)	*0.8*	−0.007 (−0.057- 0.04)	*0.8*	−0.006 (−0.056- 0.04)	*0.8*

Regression coefficients are shown as the difference in mmol/L from the reference category, adjusted for the other factors in the model.

^‡^Reference category.

Predictor variables: Social class- non-manual = social classes I, II and III non-manual, manual = social classes III manual, IV and V.

Education- at least O level, no qualifications.

Deprivation level- based on Townsend deprivation scores: < 0 = less deprived, > 0 = highly deprived.

## Discussion

In the present study, we analysed the association between three different measures of socio-economic status and different components of serum lipid levels. We observed associations between occupational social class and educational level with serum lipid levels. The relationships with socio-economic status were more evident in women than men. There were clear sex differences in terms of which socio-economic indicator was more strongly related to serum lipid concentrations and the direction of the association. In women for example, those of lower socio-economic status, either in manual social class or with low educational level had higher levels of LDL-cholesterol and triglycerides, but no such relationship existed among men. Among both men and women, educational level appeared to be the strongest correlate of lipid levels, whereas residential deprivation level was the least related of the three parameters evaluated.

The findings of our study are consistent with previous studies in which socio-economic status were associated with serum lipid levels more strongly in women than men [5, 9]. However the direction of association appears to be opposite to that reported in most studies investigating the association between socio-economic status and serum lipid levels in developed countries [3–5]. Several studies did however report findings similar to ours [6–8]. We did not find any significant association between area deprivation and serum lipids. In a study investigating the association between area and diet, Diez Roux et al. (1999) did not find any consistent association between neighbourhood median income and cholesterol intake [23] while similar results were obtained in a study which examined the influence of neighbourhood socio-economic characteristics on CHD prevalence and risk factors [24]. Smith et al. (1998) found a significant association between area deprivation and cholesterol level but this association disappeared after adjusting for individual social class [6].

Even though men in lower social groups had somewhat lower total cholesterol level, they had a more adverse lipid profile suggesting an increased risk of CHD. Though total cholesterol, triglycerides and LDL-cholesterol level were significantly lower compared to men of higher socio-economic status, their HDL-cholesterol levels were also significantly lower. In women, socio-economically disadvantaged women not only had higher total cholesterol levels, they also had more adverse lipid profiles with higher LDL-cholesterol level and lower concentration of HDL-cholesterol. A possible explanation for this difference in men and women could be due to men in manual social classes generally having more physically demanding work compared to women and this may provide a possible protective effect against elevated total cholesterol levels. Women have also been reported to have a higher rate of obesity compared to men [25] and this may reflect both lower physical activity levels or different dietary patterns. Studies have shown that women were less active than men and were less likely to participate in regular leisure physical activity compared to men [26, 27].

There may be several reasons for the influence of occupational social class in men compared to women. The first potential explanation is that it may reflect the strong association between social class and body mass index in women. There was an association between social class and serum lipids level initially, but after adjusting for body mass index the association was not statistically significant. It could also be due to possible misclassification of women’s social class by using their partner’s instead of their own, however studies have shown that women’s health outcomes are better predicted using their partner’s social class rather than their own [28, 29]. This was however in contrast with the stronger influence of educational level in women than men. A possible explanation for this may be the influence of education on eating patterns such people with higher educational level are more likely to consume less food high in saturated fat and more fruit and vegetables. We also observed sex differences in the extent to which alcohol consumption explained the association observed between socio-economic status and HDL-cholesterol level. In women, alcohol consumption did not materially change the association observed, whereas in men it did.

### Limitations

It is not likely that the association observed between socio-economic status and serum lipids is due to bias or confounding. We have accounted for possible confounders such as age, body mass index and alcohol consumption. The exclusion of individuals with missing socio-economic data is unlikely to cause bias unless they differed from those included in the study. With regard to the use of non-fasting blood samples instead of fasting blood samples, studies have shown that non-fasting lipid profiles predicted increased risk of cardiovascular events [30, 31]. The use of partner’s social class instead of women’s own did not influence the association observed as a separate analysis showed that the results did not change even when women’s own social class was used (results not shown).

## Conclusion

The findings of this study suggest that there are sex differences in the association between socio-economic status and serum lipid levels. In men, being in manual social class appears to be associated with lower serum lipid levels while alcohol consumption in men of higher socio-economic status explained the positive association between socio-economic status and HDL-cholesterol level. Low socio-economic status on the other hand was related to more adverse lipid profile in women but BMI accounted for the association observed between occupational social class and serum lipid level. This suggests that the variations in lipid profile with socio-economic status may be largely attributed to potentially modifiable factors such as obesity, physical activity and dietary intake.

### Summary box

#### What is already known?

Several studies have observed inverse relationships between socioeconomic status and cholesterol levels, others have reported higher cholesterol levels among those with higher socioeconomic status while some found no association at all. In most of these studies, socio-economic status was usually measured using only one indicator of socio-economic status.

#### What this study adds?

The findings of this study suggest that there are sex differences in the association between socio-economic status and serum lipid levels. In men, being in manual social class appears to be associated with lower serum lipid levels while alcohol consumption in men of higher socio-economic status explained the positive association between socio-economic status and HDL-cholesterol level. Low socio-economic status on the other hand was related to more adverse lipid profile in women but BMI accounted for the association observed between occupational social class and serum lipid level. This suggests that the variations in lipid profile with socio-economic status may be largely attributed to potentially modifiable factors such as obesity, physical activity and dietary intake. This has policy implications in terms of developing effective interventions at both the individual and community level.
